# Impact of Personal Experience of COVID-19 Disease on Recreational Anglers’ Attitudes and Behaviors

**DOI:** 10.3390/ijerph192416551

**Published:** 2022-12-09

**Authors:** Andrzej Robert Skrzypczak, Emil Andrzej Karpiński, Natalia Maja Józefacka, Robert Podstawski

**Affiliations:** 1 Department of Tourism, Recreation & Ecology, Institute of Engineering and Environmental Protection, University of Warmia and Mazury, Oczapowskiego St. 5, 10-719 Olsztyn, Poland; 2 Institute of Psychology, Pedagogical University of Krakow, Podchorążych 2, 30-084 Krakow, Poland

**Keywords:** recreational fishing, environmental factors, health, COVID-19, socio-economic conditions, intergroup relations

## Abstract

Background: Anglers are a large social group with access to a “relatively safe” form of recreation, that allows the opportunity to relieve stress. An important question, however, is how they did so, and to what extent their perceived COVID-19 transition status influenced decisions both in life and at the fishing site. Aim: Our study aimed to determine the dynamics of anglers’ attitudes and behaviors during the COVID-19 pandemic as a result of the different statuses of their exposure to the SARS-CoV-2 virus. We assumed that the behavior of anglers who have not experienced the disease (were not ill and not sure if ill) will be similar and, on the other hand, different from the behavior of those who have experienced COVID-19. Methods: The web-assisted interviews survey was used among 586 anglers with different COVID-19 disease experience statuses. Their pandemic behavior and activities by four age groups were studied using non-metric multidimensional scaling. Redundancy analysis has been used to identify the relationship between anglers’ life attitudes and socioeconomic and demographic factors, taking into account their preferences and involvement in fishing. Results: We have demonstrated that the behavior of anglers who have not experienced COVID-19 disease and do not present a reckless attitude toward pandemic threats, do not show significant differences from the life attitudes of the group experienced by the SARS-CoV-2 virus. These two groups comprise more than 70% of anglers. However, the rest show a lack of interest in an aware diagnosis of their health and a low level of acceptance of self-restraint in the area of direct social contact. Conclusions: Unawareness, combined with ignorance, could be a potential factor in the transmission of the virus while fishing. The behaviors of almost 30% of anglers are particularly risky when combined with a strong need to fish in the company of friends and familiar people. Anglers’ social identity should be tapped by fishery managers. Targeted educational campaigns should be aimed at groups around specific fishing spots. The need for self-limitation under the pandemic should be promoted for the benefit of the general public and to maintain the reputation of angling as a safe recreational activity.

## 1. Introduction

The SARS-CoV-2 virus as well as the COVID-19 disease caused a pandemic in the early 2020 [1]. The disease itself, as well as the restrictions that followed it, caused many social [2,3,4,5,6], health [7,8,9], and economic [10,11] problems in 2020–2021 and its negative effects are likely to be a problem for many communities for a long time to come [12]. Even the environmental effects, initially considered positive, have turned out to be rather ambivalent [13,14]. It appears that this disease will stay with us for a longer period, and despite its apparent transition to a less threatening form e.g., [15,16], we certainly should not lose our sense of caution. Nevertheless, the memories of those times will stay with us for a long time. Both restrictions and health problems, personal or infrastructural caused big impacts on every population e.g., [17]. Although the disease has mainly taken its toll on the elderly [18,19], many people of working age have also been severely or very severely affected and it has been a very traumatic experience for them, both physically e.g., [20] and psychologically e.g., [6,21].

Despite the many infections, recorded with unprecedented meticulousness, the size of the world population that has encountered this serious respiratory disease according to statistics does not exceed 8% of the world population [22]. This amount, due to data accessibility problems and the nature of the disease, is obviously higher. It is estimated that the number of asymptomatically infected people varies between 4 and 41% [23]. Consequently, accurately determining the number of people infected at least once seems to be an impossible task. Asymptomaticness, combined with the use of masks [24], vaccination [25], and poor education [26] may have been associated with a false sense of security and a lack of response to imposed restrictions or indicated good practices. However, regardless of whether the lack of illness was actual or we became asymptomatic, our perception of the reality of the pandemic may have differed. Infection status may have resulted in different perceptions of individual health issues and behaviors among other people, which were part of widely held restrictions during the pandemic e.g., [27,28].

During the pandemic, there were many restrictions imposed on the realm of sport, tourism, and recreation [28,29,30,31]. Even though some of the leisure behaviors were likely to be considered relatively safe due to their characteristics and outdoor locations [32], they were halted. This, of course, was at first, due to precaution, because in general there is always some dose of risk [33], but with good practices and some modifications, it was possible to practice this hobby without much subjective concern.

One of the forms of recreation considered safe during the pandemic was fishing [34,35,36,37]. It is a very popular form of recreation worldwide [29,38,39,40], practiced outdoors, where it is less likely to become infected [35], also due to its non-contact nature. Its characteristics, good practices, and sometimes even the law [41,42], enforce keeping a distance regardless of the potential fear of infection, as vigilance against being hooked should be constant. It is also worth mentioning in this context that anglers need space to set up their equipment and distance themselves from each other in order not to “flip” the line of the colleague next to them. Therefore, it is a form of recreation that allowed people to relax in a state of constant tension caused by both the actual possibility of being infected and the omnipresent information and media-fueled atmosphere of fear [43]. Additionally, this relaxation could take place in nature in a space that often has health-promoting and restorative properties [44] regardless of the season [45]. Anglers, as a large social group with access to a “relatively safe” form of recreation, therefore had the opportunity to relieve stress. However, fishing, despite its characteristics, has also been affected by restrictions to varying degrees [34,46].

Significant variation in public attitudes and reactions to the declared pandemic and imposed restrictions have been demonstrated by Rothmund et al. [47]. According to Alsubaie et al. [48], there are demographic factors associated with a high denial attitude. It has also been shown that withdrawal from prevention and acceptance of pandemic skepticism attitudes may pave the way for more COVID-19 cases [49,50]. Fear is inherent in the COVID-19 characteristics and is not completely manageable [51]. According to Mancini and Imperato [52] research, a sense of social belonging reduces the negative impact of anxiety on health. In fact, fear and vulnerability may increase perceptions of identity threats [53].

Since pandemic behavior can change under the influence of various factors, we decided to study it among anglers, who practice one of the safer forms of recreation and who form a certain community. Our study aimed to determine the dynamics of anglers’ attitudes and behaviors during the COVID-19 pandemic as a result of the different statuses of their exposure to the SARS-CoV-2 virus. We assumed that the behavior of anglers who have not experienced the disease, and therefore have not been ill or were not sure about contact with the disease, would coincide and be different from the behavior of those who have experienced the disease. The basis for this hypothesis is the assumption that people with direct experience of the disease will be a more homogeneous group in their attitudes and behavior. This is based on the published research results and the idea that there will be no coronasceptics in this group. Nor should it include people who believe in the virus and in their body’s extraordinary immunity, which has yet to be verified. The following research questions were formulated: (1) To what extent did the perceived status of contact with the virus and getting sick influence anglers’ decisions both in real life and in the fishing spot; (2) how the socioeconomic factors, as well as the preference and engagement factors, explain the variation in the perceptions and behaviors of anglers with different SARS-CoV-2 virus transmission status, including the age factor.

## 2. Materials and Methods

### 2.1. Design of the Survey Questionnaire and Data Collection

The survey questionnaire was based on 19 items divided into five groups and it was constructed on the Google Forms platform in two languages (English and Polish). The first question about SARS-CoV-2 virus transition status was differentiating and assigned anglers to three various tested groups: definitely had C-19 infection (hereinafter C-19_Y), definitely had not had C-19 infection (hereinafter C-19_N), and not sure if got infected (hereinafter C-19_D). 

The second group of questions was basic socio-economic data including gender, education, age, and place of residence (in thousands of inhabitants). The third part consisted of questions about engagement in angling. The data on sociodemographic matters and questions on involvement in fishing required the entry of a specified value. In other cases (gender, education) there were options to choose from. Information about preferences for fishing among others (family, friends, or alone) was collected in the 4th part of the survey. The last part stored information on perception and behavior toward angling and life attitudes during the COVID-19 period. The last two parts were measured using opinions/preferences 5-point Likert scale. This type of scale consists of answers ranked 1–5, where “1” was the equivalent of “I strongly disagree”, “5” means “I strongly agree”, and “3” means “I have no opinion, or it is difficult to determine” (neutral opinion). This type of scale is commonly used in surveys collecting opinions on all sorts of topics [54,55].

In most cases, WAI (web-assisted interviews) survey was used. This type of survey is very efficient, easy, and cheap. Moreover, it is completely anonymous and less error-prone compared to traditional questionnaires [56]. It allows fast access and analysis of the data. Survey respondents were encouraged to distribute it among their angling communities involving snowball sampling [57] (to obtain so-called “virality” [58]) with the restriction of completing the survey only once. It was made to prevent problems with the WAI survey, where we do not know who is filling out the survey and whether they are filling it out more than once. Additionally, complete sets of responses were analyzed for filling out incorrectly, incompletely, or shoddily. Those sets of questions were removed from further analyses. 

The questionnaire was distributed via social media platforms (e.g., angling associations and clubs, Facebook groups and fan pages, anglers’ discussion groups, and Internet forums) and other various popular angling websites in English and Polish. Therefore, the sample included people active on fishing social media. Given the spatial impact of the sites, groups, and profiles, it should be assumed that the survey covered mainly European residents. However, determining the response rate seems impossible due to the inability to conclude how many people actually saw the survey. Judging by the number of observers, it could have been up to 500,000 people. We also used a small portion of paper surveys (approx. 3–5%) based on the framework implemented in the web survey. They were widespread in older people angling communities that prefer this type of survey taking into account the importance of the article for the elderly. These traditional surveys were collected in a way that made it impossible to link specific results to a specific person in the spirit of complete anonymity. This data were manually added to the automatically generated WAI survey data. The questionnaire was also fully voluntary and not limited by age. The time required to complete the survey was about 5 min. The full questionnaire is available in Table S1 (Supplementary Materials). The data were collected from July to September of 2022 when the impact of C-19 restrictions on society was not considered adequate to reflect the entire population’s opinion or judgments based on its direct impact.

In total, 586 respondents provided complete and usable answers to the survey questions, including 8% (47 questionnaires) in English. This number of completed questionnaires was large enough to apply to the entire anglers population. The margin of sampling error (MoE) at confidence level α = 95% was calculated (Table S2 (Supplementary Materials)). It was ±4.05% for the whole sample and ±1.8% to ±7.9% for each socio-demographic subgroup. A rule of thumb researchers considers adequate to reflect the opinion of the entire population is an MoE of 4–8% [59,60]. 

### 2.2. Statistical Procedures

The SARS-CoV-2 virus transition status, demographic, and engagement data were examined using frequency table characteristics. The percentages of respondents with statements related to angler preferences and behavior were calculated similarly. Using a Likert scale, we assumed its non-linear distribution [61]. Consequently, we used dedicated non-parametric tests to analyze an ordinal scale data [62].

We examined the differences between the preferences and behavior of the anglers’ groups with different SARS-CoV-2 virus transition statuses by using a non-parametric Kruskal–Wallis ANOVA rank-sum test for independent samples (*p* < 0.05). Due to the large sample size, the variation in respondents’ opinions was chosen to be shown not by median but by mean and standard deviation. In identifying the preferences and pandemic life attitudes of anglers with different disease experience statuses, cases respondents who declared “I have no opinion, or it is difficult to determine it” (neutral opinion) were excluded from the analysis. This resulted in correcting the interpretive error of the mean Likert scale score, leaving respondents declaring no opinion in the analysis would have flattened the results (mean and standard deviation). Information about the number of the sample subjected to the statistical test was provided with the test result. All statistical significance tests were performed using Statistica version 13.3 software. The life attitudes of age groups of anglers (<26 y/o; 26–40 y/o; 41–65 y/o; 66–85 y/o) with different COVID-19 disease experience status, their behaviors, and activities under the pandemic were tested with the non-metric multidimensional scaling (NMDS). The Bray–Curtis distance measure, two axes, and stress formula type 2 were applied for log transformed variables [63]. The analysis was conducted using Canoco 5.11.

Redundancy analysis (RDA), as a canonical form of principal component analysis and one of the linear techniques used in socio-economic research, has been used to the identification of the relationship between anglers’ life attitudes as well as socioeconomic and demographic factors, taking into account their preferences and involvement in fishing. The usefulness of this linear ordering method is determined by the size of the standard deviation in the dataset, i.e., when the largest gradient in the dataset does not exceed 3.0 [63,64].

The RDA space was used to explain the life attitudes of four age groups of anglers with different COVID-19 disease experience statuses, including the following feelings and behaviors: fear of getting sick, attitudes toward vaccination, limitation of contact with family and friends, limitation of staying in closed and outdoor public spaces, fear of virus infection in the fishing spot, more frequent fishing during the pandemic. Anglers’ responses were compositional and had a gradient of 0.2 SD unit lengths, so a linear method better explained the data. Each variable that explained anglers’ feelings and behavior was tested for statistical significance using Monte Carlo tests (499 random permutations). Data were normalized using the log (x + 1) transformation [63]. All variables explained a significant amount of variation and were statistically significant (*p* < 0.05). The explanatory variables (sociodemographic factors, preferences, and engagement indicators) were selected based on a variance inflation factor (VIF) of less than 10. During the RDA analysis, the numbers of response data (life attitudes during the pandemic) and explanatory variables were verified each time based on the values of the correlation coefficients of the explanatory variables and VIF. The purpose of this verification was to obtain the maximum value of the percentage of the explained total variance of response data [63]. Finally, the eight explanatory variables were implemented into the ordinal space, including a preference for fishing with family; preference for fishing with friends; preference for fishing alone; distance to the most visited fishing spot; avidity expressed by the frequency of fishing; educational level; place of residence expressed in the number of inhabitants; experience expressed by years of engagement in fishing. RDA was performed using the Canoco version 5.11 software.

## 3. Results

### 3.1. Summary of Responses—Sociodemographic and Engagement Characteristics of Anglers’ Groups with Different SARS-CoV-2 Virus Transition Statuses

Respondents (*N* = 586), were divided into three groups according to their infection status. Their quantities were as follows: C-19_Y (*N* = 167), C-19_N (*N* = 246), and C-19_D (*N* = 173) which can be seen in Table S2.

The great majority of respondents (95.1%) were men. Women were most represented in the C-19_D group constituting 9.3%. Most vulnerable to COVID-19 infection group—the elderly (older than 65 y/o) represented 9.7% of the population and were most common in the not infected group (12.2%). In the C-19_D group respondents, up to 40 y/o had the highest numbers (67.6%) compared to 52.7% in C-19_Y and 45.1% in C-19_N groups. Education status was fairly distributed in all distinguished groups showing that most respondents have secondary education (42.5%). Respondents came from all sorts of different-sized settlements, most often from rural areas (23.5%) and large cities (27.6%).

Respondents’ engagement expressed by angling frequency showed that the larger part of anglers, regardless of the group, were avid and fished once a week and more often (50.2–60.7%). Only 10.6% were sporadic anglers. The C-19_Y group was least avid with about 4 days less spent on fishing during the year than other groups. The C-19_D group has the greatest proportion of the most avid anglers but also has the most anglers fishing least frequently (15.6%). It has also the greatest disproportion in typical distance traveled to the fishing site containing pro rata both the most anglers traveling for the longest distances (more than 50 km) for fishing (16.8%) and the anglers within 5 km from the most often fished spot (32.9%). However, all groups have a similar mean distance to the most often fished spot (around 21 km) Most experienced group was C-19_N with almost a third of the anglers (33%) having more than 30 years of fishing practice and mean of around 23 years of experience. More than a quarter of surveyed anglers (26.1%) were new to the activity with experience of fewer than 5 years and the C-19_D group was least experienced (about 20 years of experience).

### 3.2. Anglers’ Life Attitudes and Behavior in Relation to Personal Experience of COVID-19

No less than 42.3% of respondents preferred fishing alone (Table S3), and none of the separate groups differed significantly on this question (Table 1). Similarly, the lack of differences can be seen in questions about preferences for fishing with friends (47.4%) and family (28%). However, it should be pointed out that the C-19_Y group has a slightly higher average for fishing alone and a slightly lower average for fishing with family. The majority of respondents (66%) were not worried about contracting COVID-19 (or getting sick again) and were rather negative about it (means below 2.17). The C-19_D group was least concerned, which differed significantly from the most concerned C-19_N group. It is also noteworthy that those infected were more diverse in that matter in their opinions (SD 1.64) than the other groups (SD 1.15–1.29). Anglers as a whole were rather positive about vaccination for COVID-19, with 72.5% having at least a neutral opinion on it. Most pro-vaccination was C-19_N, while the C-19_D group was rather neutral (mean 2.96 on a 5-point scale), but was also internally the most divided (SD 1.85—highest considering all questions in the survey). This group was also less likely to limit contact with family (mean 2.44) and friends (mean 2.56) and differed significantly in its opinion on this topic from the other two groups, which were more neutral or even positive on this issue (mean 3.02–3.47). However, all groups tended to limit their activity in indoor spaces (57.7 percent and mean 3.46–3.84), with the C-19_Y limiting it to the most extent. The opposite is true for limiting outdoor activity. All groups did so rather infrequently (17.9 percent), with the C-19_D group doing it least often (mean 1.64 ± 1.25 SD). 

All groups felt overwhelmingly safe in the fishing grounds (96.1%) concerning COVID-19 virus infection with averages ranging from 1.11–1.17 and a negligible discrepancy (SD 0.44–0.61). More than a quarter of all anglers surveyed (26.8%) reported that they fished more frequently during the pandemic. Only the C-19_D group showed such a tendency (mean 3.13), and this was significantly different from the other groups, which rather disagreed with the statement about angling more often in this regard (mean 2.54–2.55). All groups were internally divided on this question (SD 1.51–1.81). The highest percentage of neutral responses was related to the question about more frequent fishing during the pandemic (36.7%), as well as the question about the preference to fish alone (30.7%) or in the presence of family (24.4%) and friends (25.4%).

**Figure 1:**
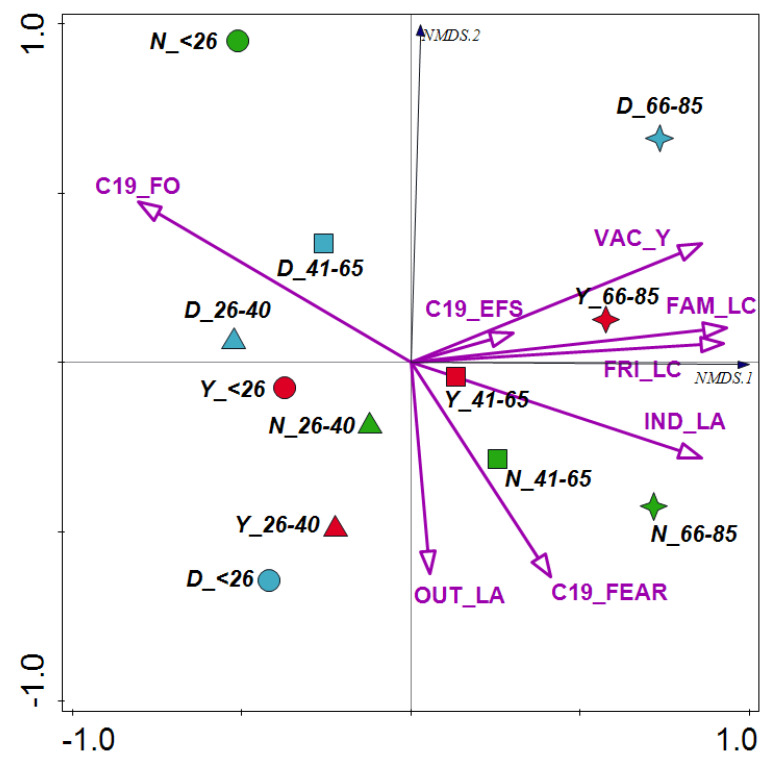
The NMDS triplot based on life attitudes of anglers with different SARS-CoV-2 virus transition statuses in four age groups. Abbreviations: *_<26*—under 26 years old (circle), *_26*−*40*—aged 26−40 years (triangle), *_41*−*65*—aged 41–65 years (square), *_66*−*85*—aged 66−85 years (star); COVID-19 infection: *Y*—“yes” (red), *N*—“no” (green), *D*—“don’t know” (blue); C19_FEAR, concerned about getting sick (or getting sick again) from COVID-19; VAC_Y, positive attitude towards vaccination against COVID-19; FAM_LC, limited contact with my family during the pandemic period; FRI_LC, limited contact with my friends during the pandemic period; IND_LA, limited indoor activities during the pandemic period; OUT_LA, limited outdoor activities during the pandemic period; C19_EFS, feel exposed to COVID-19 infection at the fishing spot; C19_FO, during the pandemic period I fished more often.

The pandemic behaviors of anglers were tested in reduced ordination space with NMDS (Figure 1). The NMDS analysis with the stress value 0.00636 has visualized the similarities and dissimilarities of life attitudes and preferences of anglers with different SARS-CoV-2 virus transition status. Most of the behaviors included in the analysis were strongly positively correlated with the NMDS 1 axis (Table 2), including reduced social contact with family (r = 0.9331) and friends (r = 0.9254), acceptance of vaccinations (r = 0.8610), limited exposure to indoor public spaces (r = 0.8602), and more frequent fishing during the pandemic (r = −8062). This axis explains 82.9% of the total variation. Pandemic behaviors most positively correlated with NMDS1 resulted in the separation of two age groups of anglers (41–65 and 66–85 years old) who had been affected by COVID-19. In contrast, the restriction of being in enclosed public spaces and fear of contracting the disease most strongly influenced the separation of the two oldest age groups of anglers who had not yet been affected by the disease. The oldest age group of anglers who were unsure about contact with the virus was separated mainly by their acceptance of vaccination. The tendency to fish more often during the pandemic (strong negative correlation with NMDS1) made it possible to separate two age groups of anglers (26–40 and 41–65 years old) who were unsure about contact with the virus and the youngest group who were not ill. In contrast, the separation in the ordination space of the remaining groups of anglers, including those aged <26 years and 26–40 years old, was due to a lower acceptance of behavior that restricted social relationships and a skeptical attitude toward vaccination against COVID-19.

### 3.3. The Background of Anglers’ Behavior with Different SARS-CoV-2 Virus Transition Status

**Figure 2:**
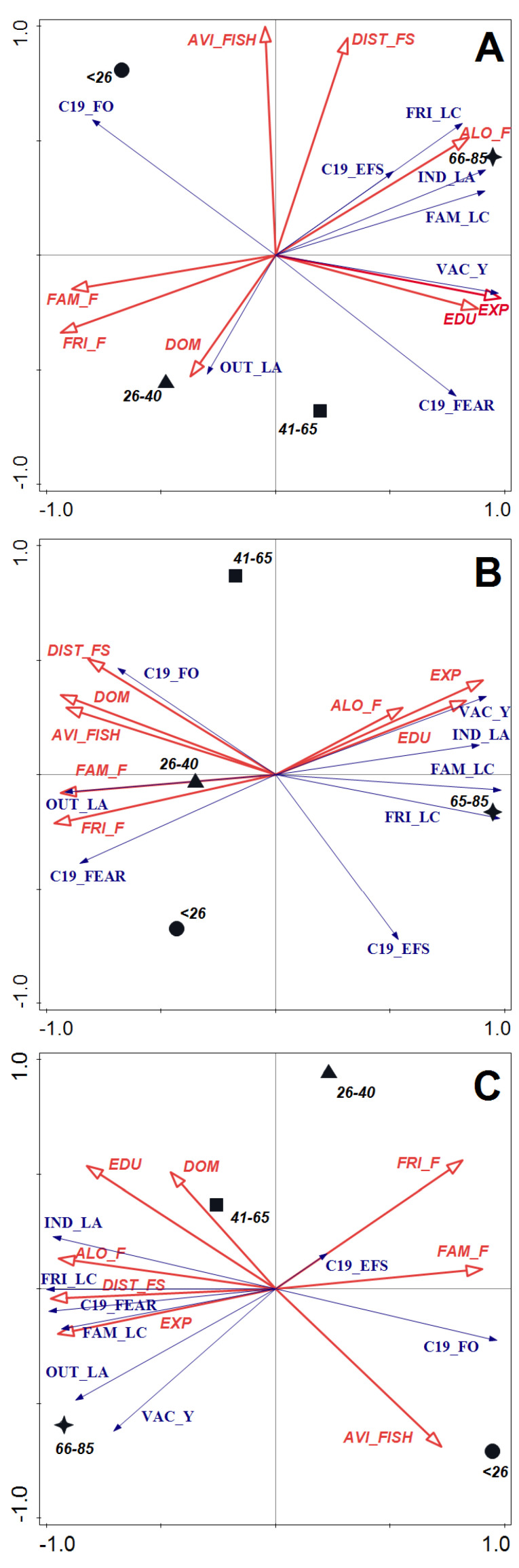
Triplot ordinal redundancy analysis (RDA) of life attitudes among age groups of anglers (**A**) infected with SARS-CoV-2 virus, (**B**) unsure of contact with the SARS-CoV-2 virus, and (**C**) not infected (response data, blue arrows) versus their angling preferences and sociodemographic factors (explanatory variables, red arrows). Abbreviations: *<26*—under 26 years old (circle), *26*−*40*—aged 26−40 years (triangle), *41*−*65*—aged 41−65 years (square), *66*−*85*—aged 66−85 years (star); C19_FEAR, concerned about getting sick (or getting sick again) from COVID-19; VAC_Y, positive attitude towards vaccination against COVID-19; FAM_LC, limited contact with my family during the pandemic period; FRI_LC, limited contact with my friends during the pandemic period; IND_LA, limited indoor activities during the pandemic period; OUT_LA, limited outdoor activities during the pandemic period; C19_EFS, feel exposed to COVID-19 infection at the fishing spot; C19_FO, during the pandemic period I fished more often; *FAM_F*, preference for fishing with family; *FRI_F*, preference for fishing with friends; *ALO_F*, preference for fishing alone; *DIST_FS*, distance to the most visited fishing spot; *AVI_FISH*, avidity expressed by the frequency of fishing; *EDU*, educational level; *DOM*, place of residence expressed in number of inhabitants; *EXP*, experience expressed by years of engagement in fishing.

The relationships between life attitudes of anglers with different SARS-CoV-2 virus transition statuses in four age groups and their angling preferences and sociodemographic variables were determined in RDA ordination (Figure 2). For each category of anglers, the correlation of all axes was significant in the Monte Carlo permutation test, including (Figure 2A) infected with SARS-CoV-2 virus: F = 3.68, *p* = 0.020 with a total variation of 30.08 and explanatory variables accounting for 80.3% of the variance; (Figure 2B) unsure of contact with the SARS-CoV-2 virus (Figure 2B): F = 6.04, *p* = 0.001 with a total variation of 37.12 and explanatory variables accounting for 82.7% of the variance; (Figure 2C) not infected: F = 5.48, *p* = 0.002 with a total variation of 32.22 and explanatory variables accounting for 81.3% of the variance (Table S4). Table S4 shows the summary of the results of the RDA including eigenvalues, correlations, and percentage of variation explained by all canonical axes.

Among anglers indicating earlier or ongoing SARS-CoV-2 infection (Figure 2A), the sum of all the canonical eigenvalues was 0.8034. The first two components of RDA explained 89.25% of the total variance of response data including the first axis accounting for 83.42%. In this group, a preference for fishing with friends and family was negatively correlated with the limitation of contact with family and friends, staying out of public indoor spaces, and fear of reinfection at the fishing spot. Such pandemic behavior was characteristic of the oldest age group and anglers who prefer to fish alone. Willingness to limit outdoor activities increased with the size of the settlement the anglers live in but decreased with fishing frequency and distance from the most visited fishing spot. Better education and length of fishing experience were positively correlated with acceptance of vaccination, but negatively toward more frequent fishing during the pandemic. Such activity was most characteristic of young anglers (<26 years old), who were least likely to show fear of re-infection and have low acceptance of vaccination. 

For anglers unsure of contact with the SARS-CoV-2 (Figure 2B), the sum of all the canonical eigenvalues was 0.8274. The first two components of RDA explained 96.42% of the total variance of response data including the first axis accounted for 88.64%. In this group, better education, length of fishing experience, and preference for fishing alone correlated positively with acceptance of vaccination and reduced activity in indoor public spaces. At the same time, however, it was negatively correlated with the reduction of outdoor activities and fear of COVID-19 disease. This fear and reduction were found to be most characteristic of anglers aged 26–40 who prefer fishing with friends and family. The oldest anglers in this group reduced social contact with family and friends during the pandemic (it was negatively correlated with the number of residents of the locality they inhabited and the high frequency of fishing. More frequent fishing during the pandemic compared to the preceding period was positively correlated with distance to the most frequently visited fishing spot). 

Among anglers who indicated that they have not been sick with COVID-19 (Figure 2C) the sum of all the canonical eigenvalues was 0.8131. The first two components of RDA explained 93.22% of the total variance of response data including the first axis accounted for 89.55%. In this group acceptance of COVID-19 vaccination and willingness to limit outdoor activities decreased with the higher need to fish with friends and family. Anglers, who fish with others were least likely to reduce social contact during the pandemic and were less fearful of COVID-19. However, they felt the greatest exposure to the infection while fishing although this perception was relatively the least intense of all described. The limitation of time spent in indoor public spaces was positively correlated with a preference for fishing alone, and negatively correlated with the frequency of fishing. Among anglers who did not contract COVID-19, we found no correlation between acceptance of vaccination and the education status or the size of the population of the locality from which they originated.

## 4. Discussion

In defining the research problem, we focused on analyzing anglers’ pandemic behaviors and the dynamics of these attitudes in relation to anglers’ different personal experiences of the disease. There was no statistically significant variation in the preference for fishing alone or in the presence of others among the three groups of anglers with different COVID-19 infection statuses. Thus, it is constant and unrelated to potentially higher morbidity among anglers who prefer to fish in a wider group. It can be assumed that even if there were some self-restrictions in this regard, they are included in the margin of statistical error and will not be permanent. This is confirmed by research on general changes in life behavior and pandemic decisions. An identified problem related to decision-making is the different perceived short-term goals (that produce a quick effect) versus long-term goals, achieved over a longer period [65,66]. It has been shown, for example, that with feelings of loneliness, social contact can destruct other goals, such as maintaining health. According to Thagard [67], pandemic constraints are undermined by the immediate short-term goals of eating, socializing, and recreation, which can lead people to discount long-term goals such as maintaining health and helping society recover from the pandemic. However, it should be noted that in the literature, the trend of decreasing numbers of people during joint recreation, including fishing is visible during the COVID-19 period [68].

Anglers who do not prefer fishing alone were more inclined to be accompanied by friends rather than family. This should be explained by the overwhelming predominance of men in the group of respondents. Differences were identified in the motivating needs of women, who, as part of social ties saturation, are more inclined than men to develop family relationships while fishing [69]. 

The majority of respondents (66%) were not worried about contracting COVID-19 (or getting sick again) and were rather negative about it (averages below 2.17). The C-19_D group was the least concerned, and statistically different from the most concerned group (C-19_N). At the same time, this group of anglers (C-19_N) had the highest acceptance of vaccination relative to the other two groups. According to Rothmund et al. [47], the fear of getting sick in social groups aware of the threat of the SARS-CoV-2 virus is linked to acceptance of restrictions and positive attitudes toward vaccination.

All anglers were positive about limiting their activities in enclosed public spaces and had no fear of infection while out fishing. The latter behavior confirms a sense of safety toward their recreational activity. The belief that angling is widely regarded as a safe form of outdoor recreation was demonstrated in studies by other authors [34,35,37].

Restrictions on staying in indoor public spaces have been imposed by lockdown policies, which many societies in Europe and around the world have experienced [70]. Therefore, it is difficult to determine unequivocally to what extent the acceptance and adherence to such pandemic restrictions was the respondents’ initiative. Against the background of other behaviors, it is reasonable to believe that it was largely forced. This is indicated by the statistically lower acceptance of voluntary self-restriction of social contact (with family and friends) and staying outdoors and in open public spaces, characteristic of anglers with unidentified disease contact status. At the same time, they were most likely to declare more frequent angling during the pandemic period. In light of previous research, such attitudes should be interpreted as a denial of pandemic threats [71]. The background to such attitudes is complex, and coronascepticism manifests itself in ignoring the threat and denying the sense of the restrictions being introduced [72,73]. In our study, the group not interested in being diagnosed with COVID-19 accounted for just over 29% of respondents. This figure is consistent with the findings of Rothmund et al. [47], who identified pandemic denial in 27% of respondents.

The greatest skepticism about the restrictions and limitations introduced concerning the pandemic, and a tendency to frequent angling, was shown by certain age groups: 26–40 and 41–65 y/o, who were unsure of their exposure to the virus, as well as the youngest who were sick or not sick (<26 years old). At the same time, these groups of anglers manifested the lowest rate of acceptance of vaccination. According to Rothmund et al. [47], pandemic denial and unwillingness to be diagnosed and vaccinated is the effect of doubt. It mainly affects middle-aged people with cognitive characteristics and lower levels of education. In contrast, in the youngest part of society, it is the effect of disregard. Among other things, it characterizes those who are less educated and convinced of the good state of their health, and who reject the possibility of contracting the SARS-CoV-2 virus, Rothmund et al. [47]. In contrast, the greatest acceptance of self-limiting behavior during the pandemic was shown by anglers who had already been affected by the disease and were over 40 years of age with a particular emphasis on the oldest (66–85 years). Similar tendencies to respect pandemic restrictions were shown by anglers who were confident of not being affected by the virus and belonged to the same age groups (age over 40). If the C-19_D group should be regarded as coronasceptics, its oldest representatives (aged 66–85) showed several attitudes consistent with the behavior of the oldest anglers who had already experienced the disease. In summary, the greatest acceptance of COVID restriction and the least coronascepticism were exhibited by the oldest anglers. An interpretation of such attitudes may be that fitness and health decline with age, while fear of illness increases. Supporting this thesis are the findings of Kim and Kim [74]. 

Similar findings can be also seen in other studies [75], but it should be remembered that not every study approves such information [76,77]. Maybe it should be concluded that this kind of behavior is typical, but also connected to other factors such as for example good medical care in the country where the survey is conducted. Another group showing pro-restrictive behavior is women, Wang et al. [75], the most common in the C-19_D group. This group is most internally divided one having the greatest SD and disproportions because it has a group of coronasceptics as well as most pro-restrictive women and elderly. Several behaviors and opinions connected to the limitation of contacts and outdoor activities show that the similarity between C-19_Y and C-19_N groups is greater than between C-19_N and C-19_D. It could be assumed that the C-19_Y and C-19_N groups are people who diagnose themselves and are more aware of the threat. Moreover, the greater acceptance of self-limiting behavior during the pandemic was shown by anglers who had already been affected by COVID-19 which may be related to greater concern for society as a whole. What seems to be important is how people perceive themselves and their relative safety and it is shown in studies, e.g., [78,79] that personal perception is very important. Even in angling, during which, as the results of many works show [34], recreationists did not limit themselves to a large extent in relation to other activities because of their sense of relative safeness [37].

The lack of fear of contracting the disease, along with a belief in safety in fishing and low support for limiting outdoor and open-air activities, favoring the preservation of current activity levels of anglers who are aware of pandemic threats and are confident about their COVID-19 contact status. Referring to Caputo & Reichert’s [80] study, they may view their recreational activities as increasingly important to their mental health due to COVID-19-related stressors, or as an antidote to feelings of isolation due to restrictions on their freedom to gather and travel. A similar belief, coupled with negative attitudes toward covid restrictions among non-diagnosing coronasceptics, manifests itself in a potential increase in their angling activity levels. If this group of anglers, as well as the youngest anglers in the groups aware of their disease status (<26 years old), prefer to fish other than alone, they are potentially unconsciously contributing to the spread of the disease while fishing as well. It has been shown that withdrawal from prevention and acceptance of pandemic skepticism attitudes may pave the way for more COVID-19 cases [49,50].

Among anglers who were already ill, the tendency to limit activities in open public spaces and outdoors is strongly correlated with the population density of their place of residence. Such regularities were not found for the other two groups of anglers. This is confirmed by the findings of Rothmund et al. [47]. They showed that awareness of pandemic risks promotes the manifestation of preventive attitudes. It is also consistent with the results of a study [81], which shows that the risk of virus transmission increases in larger urban centers. 

Our study found that among anglers with unidentified virus contact status, more frequent angling during the pandemic was positively correlated with: traveling greater distances to the most frequented fishing grounds, living in larger population centers, and having greater angling activity. Only in villages and small towns C-19_D group was slightly more inclined to limit social contact. Considering the aversion to limiting social contact in the larger urban environments that are more at risk of virus transmission, and the reluctance of these anglers to diagnose the state of their health, the behavior of this group makes it a potential danger to themselves and others. So, it would be appropriate to consider how to influence a change in this behavior. 

Public education is undoubtedly a factor in the acceptance of health-promoting attitudes during a pandemic [82]. However, in the case of COVID-19, this is not obvious and is not necessarily directly linked to education. According to Alsubaie et al. [48], there are demographic factors associated with a high denial attitude, including, among others a low level of education. However, Cvetković et al. [83] empirically showed that education level does not affect preventive measures. It was found that mistrust of science was strongly negatively associated with the adoption of preventative strategies, such as physical distancing, and with adherence to public health experts’ guidance concerning COVID-19 [84]. It has indeed also been shown that mistrust is also a negative correlate of willingness to be vaccinated [85]. In our study, higher levels of education in each group of anglers were positively correlated with acceptance of vaccination and understanding of the need to reduce social contact. Therefore, it is reasonable to believe that the basis of pandemic behavior is more complex, as confirmed by other studies [71]. Ajzen [86] found that human attitudes have several components, including a behavioral component that is crucial to understanding the relationship between attitude and behavior. In the social learning process, people adapt attitudes from others, i.e., new information and forms of behavior from others, which is not necessarily directly related to education [87]. This theory may explain that one of the foundations for the consolidation of coronascepticism is the similar attitudes of family members, friends, and colleagues. Lavorga and Myles [88] studied how individual and social factors were related to the science of denial mechanism, which was analyzed using social learning theory. Human individuals tend to adjust their attitudes to resemble their reference groups [89]. 

In light of this research, it is important to consider that membership in an angling community can, to some extent, shape and reinforce pandemic attitudes. This will include both positive (safe) and undesirable behaviors. In light of our results, regardless of the status of one’s personal experience of COVID-19 disease, a preference for fishing in the company of family and/or friends is negatively correlated with behavior that reduces social contact with family and friends during a pandemic. This kind of behavior is common for people heavily connected to social ties. According to Breakwell [53], behavioral strategy is directly related to individual risk assessment. At the same time, from Mancini and Imperato [52] research, the COVID-19 strategy of excessive risk-raising is associated with poorer health, while a feeling of social belonging reduces the negative impact of anxiety on health. Therefore, in the context of our study, maintaining social identity by fishing together can be interpreted as one of the strategies for dealing with pandemic stress. Despite the reduction in the number of people co-recreating [68], according to other research [69], there is a portion of people in the society for whom the social aspect of fishing is so important that they would probably sooner stop the hobby than give up fishing with others. The strong need to saturate social ties, realized during recreational behavior, is also evident in the reluctance to reduce social contact during the pandemic [65,67]. 

## 5. Conclusions 

Our study shows that the behavior of anglers who have not experienced COVID-19 disease, but at the same time do not present a reckless attitude toward pandemic threats, do not show significant differences from the life attitudes of the group experienced by the SARS-CoV-2 virus. These two groups make up the vast majority of the angling community (more than 70%). Middle-aged and older anglers present the greatest self-aware acceptance of the need to identify their condition and limit their social contacts. This accounts for the perception of angling as a safe form of outdoor recreation. At the same time, it should be concluded that the coronasceptic attitudes of anglers are an issue that can disrupt this fairly widespread belief, although we did not find that a preference for fishing in a larger group would translate into greater morbidity.

Nevertheless, an identified problem is this group’s lack of interest in an aware diagnosis of their health and low level of acceptance of self-restraint in the area of direct social contact. These behaviors are particularly risky when combined with a strong need to fish in the company of friends and familiar people. In our study, the group of anglers meeting these criteria in various configurations and varying intensity of attitudes ranged from 17% to 29% of respondents. Unawareness, combined with ignorance, could be a potential factor in the transmission of the virus while fishing. Of course, this is an area for further research. After all, it is to be expected that restrictive policies and anglers’ behavior toward the COVID-19 pandemic will undergo dynamic changes influenced by many factors.

The pandemic has not caused a drastic decline in the involvement in angling. It does not seem to have changed the approach to fishing in most anglers. This applies especially to the most active ones and those seeking social ties in fishing. It does not seem that angling should be banned top-down and without public consultation for health reasons. Due to the nature of angling, it is considered relatively safe. In addition, it is not associated with the fear of the possibility of infection in the fishing spot. Therefore, policymakers must weigh the disadvantages of restrictions against safety gained for society’s health at the international and regional levels. Taking advantage of anglers’ social identity, managers of fishing spots should target educational and advertising campaigns to communities directly related to their grounds. Education should be based on promoting safe behavior and self-limitation in daily life during pandemics. It should raise awareness of the collective benefits of upholding the image of angling as a safe recreational activity.

## 6. Study Limitations

This study has several limitations which should be addressed in future research. The group of respondents was dominated by men, who may perceive the issues of pandemic risks and restrictions differently. We also did not ask whether the professional activity of anglers is carried out in contact with other people. This could affect the higher risk of infection in the work environment and disturb the conclusion of higher morbidity when there is a simultaneous preference for fishing in a larger group. We did not ask in detail about perceptions of risk and feelings of social identity during a pandemic. We did not ask about political preferences and issues of faith and religion. Furthermore, we do not know to what extent the accumulation of COVID-19 stress factors may influence depressive states and decisions to abandon recreational activities among anglers. There is a lack of information about susceptibility to anxiety and the causes of this fear. Vulnerability to pandemic stress may be due to many social, economic, and health factors that we did not ask about. In light of the available survey results, these may influence perceptions of the COVID-19 pandemic, attitudes toward restrictive policies, or the adoption of coronasceptic attitudes. In the future, the group that experienced COVID-19 disease would need to be defined in more detail and it would also be worthwhile to learn more about the infection. Namely, whether the disease had a mild or severe trauma in those who survived. This trauma may have been a key element in the decision to change behavior, and future research on this issue should explore this area of research.

## Figures and Tables

**Table 1:** The life attitudes and preferences of anglers with different SARS-CoV-2 virus transition statuses (Infection—I have already been affected by COVID-19: “yes”- C-19_Y; “no”- C-19_N; “don’t know”- C-19_D) (*N* = 586). Values with various superscripts are significantly different using the non-parametric Kruskal-Wallis test.

Anglers’ Characteristics	Mean Score (±SD) in Groups with Different SARS-CoV-2 Virus Transition Status *		
	C-19_Y	C-19_D	C-19_N
I fish alone	3.44 ± 1.28	3.25 ± 1.54	3.20 ± 1.61
I fish with my family	2.50 ± 1.42	2.88 ± 1.72	2.54 ± 1.59
I fish with my friends	3.56 ± 1.37	3.55 ± 1.59	3.47 ± 1.73
**^1^** I am concerned about getting sick (or getting sick again) from COVID-19	**^AB^** 1.80 ± 1.29	**^B^** 1.56 ± 1.15	**^A^** 2.16 ± 1.64
**^2^** I have a positive attitude toward vaccination against COVID-19	**^A^** 3.52 ± 1.61	**^A^** 2.96 ± 1.85	**^B^** 4.20 ± 1.51
**^3^** I have limited contact with my family during the pandemic period	**^A^** 3.02 ± 1.55	**^B^** 2.44 ± 1.70	**^A^** 3.08 ± 1.61
**^4^** I have limited contact with my friends during the pandemic period	**^A^** 3.23 ± 1.56	**^B^** 2.56 ± 1.77	**^A^** 3.47 ± 1.62
I have limited indoor activities during the pandemic period	3.55 ± 1.44	3.46 ± 1.64	3.84 ± 1.55
**^5^** I have limited outdoor activities during the pandemic period	**^A^** 2.00 ± 1.28	**^B^** 1.64 ± 1.25	**^A^** 2.07 ± 1.47
I feel exposed to COVID-19 infection at the fishing spot	1.17 ± 0.61	1.11 ± 0.57	1.13 ± 0.44
**^6^** During the pandemic period I fished more often	**^A^** 2.55 ± 1.51	**^B^** 3.13 ± 1.66	**^A^** 2.54 ± 1.81

Notes: Values with various superscripts (**^A^**^,^**^B^**) are significantly different… using the non-parametric Kruskal-Wallis test; * mean score excluding “I have no opinion, or it is difficult to determine it” (neutral opinion); **^1^** H_(N=468,df=2)_ = 13.66, *p* = 0.0011; **^2^** H_(N=486,df=2)_ = 51.23, *p* < 0.0000; **^3^** H_(N=450,df=2)_ = 13.81, *p* = 0.0010; **^4^** H_(N=462,df=2)_ = 19.74, *p* = 0.0001; **^5^** H_(N=447,df=2)_ = 13.75, *p* = 0.0010; **^6^** H_(N=351,df=2)_ = 4.09, *p* = 0.0445.

**Table 2:** The response of life attitudes of anglers with different SARS-CoV-2 virus transition statuses in four age groups to NMDS 1 and NMDS 2.

Anglers’ Characteristics	NMDS 1	NMDS 2
C19_FEAR—concerned about getting sick from COVID-19	0.4151	−0.6328
VAC_Y—positive attitude towards vaccination against COVID-19	0.8610	0.3505
FAM_LC, limited contact with my family during the pandemic period	0.9331	0.1042
FRI_LC, limited contact with my friends during the pandemic period	0.9254	0.0571
IND_LA, limited indoor activities during the pandemic period	0.8602	−0.2821
OUT_LA, limited outdoor activities during the pandemic period	0.0563	−0.6243
C19_EFS, feel exposed to COVID-19 infection at the fishing spot	0.3026	0.0883
C19_FO, during the pandemic period I fished more often	−0.8062	0.4741

## Data Availability

The data that support the findings of this study are available upon request from the corresponding author, and are in the Polish language.

## Supplementary Materials

The following supporting information can be downloaded at: https://www.mdpi.com/article/10.3390/ijerph192416551/s1. Table S1: The full survey questionnaire. Table S2: Sociodemographic and engagement characteristics of the surveyed anglers’ groups with different SARS-CoV-2 virus transition status (Infection—I have already been affected by COVID-19: “yes”- C-19_Y; “no”- C-19_N; “don’t know”- C-19_D) with the number and percentage of respondents (*N* = 586). Table S3. Anglers’ preferences and pandemic behavior in the opinion of the surveyed respondents (*N* = 586). Table S4. Summary statistics for RDA of anglers’ life attitudes with different SARS-CoV-2 virus transition status (response data) versus their angling preferences and sociodemographic factors (explanatory variables, VIF* < 10).
