# Supplementary material for: Impact of Personal Experience of COVID-19 Disease on Recreational Anglers’ Attitudes and Behaviors
Source: Int J Environ Res Public Health. 2022 Dec 9;19(24):16551. doi: 10.3390/ijerph192416551 (PMC9779481; doi:10.3390/ijerph192416551)
Supplement: Supplementary file 1 [file ijerph-19-16551-s001.zip › ijerph-2066983-supplementary.pdf]

Table S1. The full survey questionnaire.

No.	Question	Options				
	**I. SARS-CoV-2 virus transition status**					
1	Infection - I have already been affected by Covid-19	Yes		No		Don't know
	**II. Sociodemographic data**					
1	Gender	Male			Female	
2	Education	Primary		Secondary		Higher
3	Age	Type in the correct value according to your best knowledge				
4	Domicile – a place of residence (in thousands of inhabitants)					
	**II. Engagement in angling**					
1	Experience - how long have you been fishing? (in years)	Type in the correct value according to your best knowledge				
2	Avidity - how often do you fish? (days in a year)					
3	Distance - what is the distance you travel to your most visited fishing spot? (in kilometers)					
	**III. Preferences in angling** (how much do you agree with the statement?)*					
1	I fish alone	1	2	3	4	5
2	I fish with my family	1	2	3	4	5
3	I fish with my friends	1	2	3	4	5
	**IV. Perceptions and behaviors towards angling and life attitudes during the SARS-CoV-2 virus pandemic** (how much do you agree with the statement?)*					
1	I am concerned about getting sick (or getting sick again) from Covid-19	1	2	3	4	5
2	I have a positive attitude toward vaccination against Covid-19	1	2	3	4	5
3	I have limited contact with my family during the pandemic period	1	2	3	4	5
4	I have limited contact with my friends during the pandemic period	1	2	3	4	5
5	I have limited indoor activities during the pandemic period	1	2	3	4	5
6	I have limited outdoor activities during the pandemic period	1	2	3	4	5
7	I feel exposed to Covid-19 infection at the fishing spot	1	2	3	4	5
8	During the pandemic period, I fished more often	1	2	3	4	5

*If you do not agree with the statement, please circle "1" (Strongly disagree) or "2" (Disagree). If you agree with the statement, please circle "5" (Strongly agree) or "4" (Agree). If you do not have an opinion on a given topic or it is difficult to determine it, then please circle "3" (I have no opinion - neutral).

Table S2. Sociodemographic and engagement characteristics of the surveyed anglers' groups with different SARS-CoV-2 virus transition status (Infection - I have already been affected by Covid-19: “yes”- C-19_Y; “no”- C-19_N; “don’t know”- C-19_D) with the number and percentage of respondents (N=586).

CHARACTERISTICS	C-19_Y		C-19_N		C-19_D		MoE*
	N	%	N	%	N	%	[%]
**I. SOCIODEMOGRAPHIC DATA**							
1. Gender							
Female	4	2.4	9	3.7	16	9.3	±7.9
Male	163	97.6	237	96.3	157	90.7	±1.8
2. Age (years)							
Less than 25	27	16.2	27	11.0	27	15.6	±7.5
26-40	61	36.5	84	34.1	92	52.0	±6.3
41-65	66	39.5	105	42.7	42	24.3	±6.5
66-85	13	7.8	30	12.2	14	8.1	±7.7
3. Education							
Primary/vocational school	29	17.4	39	15.9	33	19.1	±7.4
Secondary	75	44.9	99	40.2	75	43.3	±6.1
Higher	63	37.7	108	43.9	65	37.6	±6.3
4. Domicile (in thousands of inhabitants)							
Village	39	23.3	63	25.6	36	20.8	±7.1
Town to 5	25	15.0	18	7.3	14	8.1	±7.7
A city 5 to 25	28	16.8	39	15.9	48	27.8	±7.3
A city 26-100	27	16.2	51	20.7	36	20.8	±7.3
A city over 100	48	28.7	75	30.5	39	22.5	±6.9
**II. ENGAGEMENT IN ANGLING**							
1. Experience (years)							
Less than 5 years	21	12.6	21	8.5	17	9.8	±7.7
5-10 years	18	10.8	48	19.5	30	17.3	±7.4
11-20 years	39	23.3	42	17.1	48	27.8	±7.2
21-30 years	45	26.9	54	22.0	48	27.8	±7.0
31-40 years	24	14.4	36	14.6	9	5.2	±7.6
More than 40 years	20	12.0	45	18.3	21	12.1	±7.5
2. Avidity (frequency of angling)							
A few times a year	17	10.2	18	7.3	27	15.6	±7.7
A dozen or so times a year	30	18.0	33	13.4	17	9.8	±7.5
About 2-3 times a month	36	21.6	66	26.8	24	13.9	±7.2
About once a week	48	28.7	66	26.8	63	36.4	±6.8
A few times a week	36	21.5	63	25.7	42	24.3	±7.1
3. Distance (to most often fished spot in km)							
Up to 5	33	19.8	63	25.6	57	32.9	±7.0
6-10	39	23.3	39	15.9	27	15.6	±7.3
11-30	54	32.3	75	30.5	45	26.0	±6.8
31-50	18	10.8	36	14.6	15	8.7	±7.6
51-70	11	6.6	18	7.3	15	8.7	±7.8
More than 70	12	7.2	15	6.1	14	8.1	±7.8

* MoE – Margin of sampling error (%) at 95% confidence interval

Table S3. Anglers' preferences and pandemic behavior in the opinion of the surveyed respondents (N=586).

Characteristic	The response rate on the Likert scale*									
	1		2		3		4		5	
	N	%	N	%	N	%	N	%	N	%
1. I fish alone	76	13.0	82	14.0	180	30.7	141	24.0	107	18.3
2. I fish with my family	149	25.4	130	22.2	143	24.4	73	12.5	91	15.5
3. I fish with my friends	83	14.2	76	13.0	149	25.4	106	18.1	172	29.3
4. I am concerned about getting sick (or getting sick again) from Covid-19	306	52.2	81	13.8	98	16.7	37	6.4	64	10.9
5. I have a positive attitude towards vaccination against Covid-19	130	22.2	31	5.3	80	13.6	79	13.5	266	45.4
6. I have limited contact with my family during the pandemic period	161	27.5	70	11.9	116	19.8	133	22.7	106	18.1
7. I have limited contact with my friends during the pandemic period	145	24.7	55	9.4	104	17.8	135	23.0	147	25.1
8. I have limited indoor activities during the pandemic period	94	16.0	52	8.9	102	17.4	139	23.7	199	34.0
9. I have limited outdoor activities during the pandemic period	270	46.1	92	15.7	119	20.3	61	10.4	44	7.5
10. I feel exposed to Covid-19 infection at the fishing spot	511	87.2	52	8.9	6	1.0	7	1.2	10	1.7
11. During the pandemic period I fished more often	139	23.7	75	12.8	215	36.7	66	11.3	91	15.5

* Typical 5-point Likert scale, where "1" is the equivalent of "I strongly disagree" and "5" means "strongly agree" with "3" meant "I have no opinion, or it is difficult to determine it" (neutral opinion)

Table S4. Summary statistics for RDA of anglers' life attitudes with different SARS-CoV-2 virus transition status (response data) versus their angling preferences and sociodemographic factors (explanatory variables, *VIF** <10).

SARS-CoV-2 virus transition status of anglers	**AXES**	**1**	**2**	**3**	**4**	**Total variance**
YES	Eigenvalues:	0.7721	0.0618	0.0449	0.0102	1.000
	Pseudo-canonical correlation	0.9224	0.8801	0.9042	0.8310	
	Cumulative percentage variance					
	of response data:	77.21	83.38	87.88	88.90	
	of fitted response data:	83.42	89.25	92.53	97.87	
	Sum of all eigenvalues					1.0000
	Sum of all canonical eigenvalues					0.8034
DON'T KNOW	Eigenvalues:	0.8672	0.0439	0.0290	0.0098	1.000
	Pseudo-canonical correlation	0.9631	0.9041	0.8261	0.8883	
	Cumulative percentage variance					
	of response data:	86.72	91.11	94.01	94.99	
	of fitted response data:	88.64	96.42	99.11	99.67	
	Sum of all eigenvalues					1.0000
	Sum of all canonical eigenvalues					0.8274
NO	Eigenvalues:	0.8784	0.0353	0.0194	0.0061	1.000
	Pseudo-canonical correlation	0.9313	0.8971	0.8372	0.8455	
	Cumulative percentage variance					
	of response data:	87.84	91.37	93.31	93.92	
	of fitted response data:	89.55	93.22	96.36	98.44	
	Sum of all eigenvalues					1.0000
	Sum of all canonical eigenvalues					0.8131

* variance inflation factor
